# The Application of Precision Medicine in Structural Heart Diseases: A Step towards the Future

**DOI:** 10.3390/jpm14040375

**Published:** 2024-03-30

**Authors:** Grigorios Chrysostomidis, Anastasios Apostolos, Amalia Papanikolaou, Konstantinos Konstantinou, Grigorios Tsigkas, Antigoni Koliopoulou, Themistokles Chamogeorgakis

**Affiliations:** 1 Second Department of Adult Cardiac Surgery—Heart and Lung Transplantation, Onassis Cardiac Surgery Center, 176 74 Athens, Greece; gregory.chrisostomidis@gmail.com (G.C.); koliopoulou.a@gmail.com (A.K.); themis65@hotmail.com (T.C.); 2 First Department of Cardiology, National and Kapodistrian University of Athens, Hippocration General Hospital, 115 27 Athens, Greece; amaliapap22@gmail.com; 3 Royal Brompton and Harefield Hospitals, Guy’s and St Thomas’ NHS Foundation Trust, London 26504, UK; k.konstantinou@rbht.nhs.uk; 4 Department of Cardiology, University Hospital of Patras, 265 04 Patras, Greece; gregtsig@hotmail.com

**Keywords:** cardiovascular surgery, interventional cardiology, precise medicine, individualization, 3D printing

## Abstract

The personalized applications of 3D printing in interventional cardiology and cardiac surgery represent a transformative paradigm in the management of structural heart diseases. This review underscores the pivotal role of 3D printing in enhancing procedural precision, from preoperative planning to procedural simulation, particularly in valvular heart diseases, such as aortic stenosis and mitral regurgitation. The ability to create patient-specific models contributes significantly to predicting and preventing complications like paravalvular leakage, ensuring optimal device selection, and improving outcomes. Additionally, 3D printing extends its impact beyond valvular diseases to tricuspid regurgitation and non-valvular structural heart conditions. The comprehensive synthesis of the existing literature presented here emphasizes the promising trajectory of individualized approaches facilitated by 3D printing, promising a future where tailored interventions based on precise anatomical considerations become standard practice in cardiovascular care.

## 1. Introduction

According to The National Human Genome Research Institute, precision medicine is defined as “an emerging practice of medicine that with the aid of artificial intelligence uses an individual’s genetic profile to guide clinical decisions made in regard to the prevention, diagnosis, and treatment of disease”. More specifically, it incorporates the integration of genetic risk biomarkers and pharmacogenetics in order to develop customized treatments. This is often achieved through the implementation of molecular mechanisms, which is often denoted as personalized medicine [1,2].

While genetics and pharmacogenetics might not have direct application in cardiac surgery or interventional cardiology, personalized structural heart disease interventions, either surgical or percutaneous, include the consideration of predefined variables, thus enabling the precise tailoring of interventions to align with the special requirements and features of each patient [3,4,5]. This revolutionary shift has had a profound impact on the fields of interventional cardiology and cardiovascular surgery and especially the area of structural diseases, thus extending their capabilities and outcomes. Embracing the idea of personalized interventions empowers cardiovascular surgeons and interventional cardiologists to provide meticulously customized treatment strategies that consider the specific anatomical, physiological, and genetic traits of their patients. This not only improves the accuracy and efficacy of interventions but also contributes to enhancing patient outcomes and overall healthcare quality.

**Figure 1:**
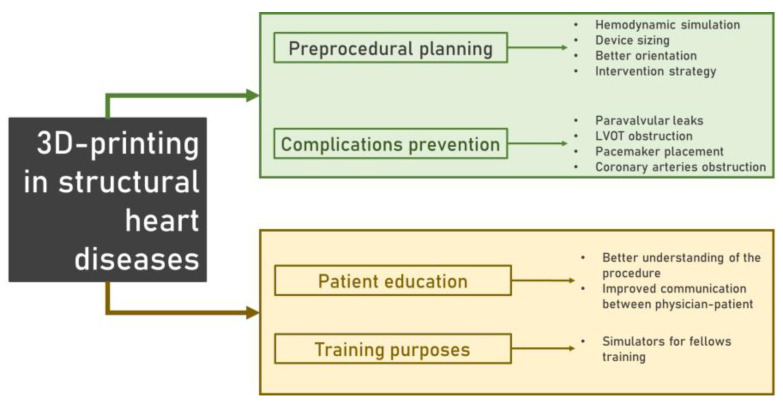
The role of 3D printing in structural heart diseases.

Three-dimensional (3D) printing remains the cornerstone of personalized medicine in surgery [6,7]. It is a digital-to-physical fabrication technique that has seen rapid integration into cardiovascular surgery. It enhances visualization of anatomical details and facilitates precision procedures. In the field of cardiovascular surgery, 3D printing has been explored for preparing well-tolerated treatments and offers substantial insights into the accuracy of surgical reconstruction in intricate cardiovascular anatomy. The technology’s utility extends to guiding both surgical and transcatheter procedures, thus contributing to improved outcomes and individualized patient care. This synthesis highlights the transformative impact of 3D printing on clinical scenarios, emphasizing its crucial role in advancing cardiac care. The applications of 3D printing expand on the assessment of procedural feasibility, device selection, prediction of outcomes, and post-procedural follow-up [8,9,10]. More specifically, in the current literature, 3D printing is utilized primarily in the preoperative planning of valvular disease and left atrial appendage closure. Apart from that, its implementation may be beneficial for educational purposes and device evaluation experiments [6,11] (Figure 1).

Imaging modalities like echocardiography, cardiac tomography, and cardiac magnetic resonance play a vital role in the utilization of 3D printing for cardiovascular applications. Echocardiography provides real-time, high-resolution images of cardiac structures, facilitating the creation of accurate 3D models [12]. CT imaging offers detailed anatomical data with excellent spatial resolution, which are crucial for complex cardiac reconstructions in 3D space [13]. CMR complements this by providing functional and tissue-specific information, thus enhancing the fidelity of 3D-printed cardiac models [12]. Through the integration of these modalities, clinicians can produce patient-specific anatomical replicas, thereby optimizing surgical planning, procedural simulation, and device customization in cardiovascular medicine.

Aρχή φόρμας

The aim of this review is to summarize the current literature regarding the personalized approaches of interventional cardiology and cardiac surgery in structural heart diseases, to analyze the existing data, and to shed light on future applications of personalized medicine in the field of structural heart treatment. 

## 2. Valvular Heart Disease

Valvular heart disease has become a prominent contributor to cardiovascular morbidity and mortality on a global scale, with projections indicating a further increase in its disease burden in the upcoming decades. Valvular heart disease includes stenosis or regurgitation; stenosis leads to elevated pressures upstream and inadequate pressure downstream, while regurgitation allows backward flow and pressure equalization [14,15,16].

Prevalent globally are distinct valve pathologies, namely rheumatic heart disease, aortic stenosis, mitral regurgitation, and aortic regurgitation [15]. A current observation is the increased prevalence of aortic stenosis in developed countries [15]. Diseases affecting the aortic valve account for 61% of mortality attributed to valvular heart disease, while the mortality for diseases of the mitral valve is lower (15%) [17]. Despite a shared bacterial etiology, infective endocarditis demonstrates a higher incidence in the elderly and is disproportionately prevalent in developed countries [18]. Although women represent a significant proportion of valvular heart disease cases globally [19,20], their underrepresentation in landmark studies shaping treatment guidelines has been a reality [19,20], accompanied by reported suboptimal postoperative outcomes [19,20].

### 2.1. Aortic Valve

Aortic valve disease, mainly stenosis and secondly regurgitation, remains the main type of valve disease with the higher incidence [14,15,16]. Aortic valve pathologies exhibit a well-established correlation with advanced age and chronic cardiovascular conditions, which is in contrast to rheumatic heart disease, an infectious complication primarily associated with crowded living conditions and constrained healthcare access [14,15,16]. For decades, the only treatment for aortic valve stenosis was surgical replacement. In the 2000s, Alan Cribier and his colleagues introduced transcathether aortic valve implantation (TAVI), a catheter-based replacement for the aortic valve using bioprosthetic prosthesis [21]. During the first years, TAVI was performed exclusively in very high-risk patients unsuitable for cardiac surgery. Nowadays, TAVI has gained significant ground and is also performed in younger patients with moderate or even low-risk surgical risk [22]. 

Whether a personalized or a general approach in patients with severe aortic stenosis is more optimal remains unclear. Contemporary medical device development employs mathematical and computer modeling, specifically finite element analysis (FEA), for in-depth analysis of organ–implant interaction. In TAVI modeling, two FEA approaches exist: generalized, utilizing averaged aortic root geometry or generic models, and patient-specific, relying on CT- or MRI-aided 3D reconstruction of a single patient’s aortic root [23]. Ovcharenko et al. showed that in specific cases, the generalized approach matches the accuracy of patient-specific modeling in TAVI. Due to its cost-effectiveness, the authors advocate for the generalized approach. Additionally, they propose that a higher level of detail enhances reproducibility in patient-specific modeling [23]. 

When performing preoperative planning of TAVR, the printed anatomy ranges from only the aortic annulus or aortic arch/aorta to more complex anatomical configurations with different anatomical structures in one print, including outflow tracts and heart chambers [10,11,24]. Moreover, preoperative TAVI simulation with the initiation of Computational analyses using finite element could be a helpful tool that has the potential to assist medical professionals in choosing the appropriate size, type, and placement of the TAVI device for each patient, aiming to minimize complications and enhance patient safety during the introduction of new valves. Apart from that, this framework could aid in designing new patient-specific devices and testing them virtually in realistic human implantation sites before resorting to animal testing, which could reduce workflow costs and avoid animal experiments [25,26]. 

Conduction disturbances following both SAVR and TAVI remain a frequent complication [27,28,29]. Several factors have been associated with higher incidence, such as displacement of calcification and deep positioning of the stented prosthesis [27,28,29]. Rocatello et al. demonstrated the clinical significance of 3D reconstruction of the aortic root, revealing a notable reduction in post-procedural conduction block incidence [30]. Utilizing a 3D-printed model of aortic stenosis with calcification, they applied balloon dilation to predict the migration direction [30]. Additionally, different stent types of valve prostheses were employed to anticipate conduction block incidence, with the investigators simulating different release positions to explore the impact of calcification on each particular deployment positioning [30]. Haghiashtiani et al. took a step further in exploring the capabilities of the 3D models [31]; they integrated an internal sensor array within the aortic root, showcasing the feasibility of introducing dynamic functionalities into these models [31]. Through the utilization of CT images, two patient-specific aortic root models were created, encompassing in detail the anatomical cardiac structures [31]. The study demonstrated the accuracy of 3D-printed models in replicating patient anatomy and behavior, as validated by postoperative data at various cardiac phases. Hemodynamic analysis emphasized the potential value of 3D-printed models with internal sensors in predicting conduction disturbances and providing quantitative assessments of pressure changes in relation to different valve sizes [31]. Moreover, Galli et al. reported that Machine learning (ML), by combining statistical and mechanistic modeling, could successfully predict conduction abnormalities following TAVI, thus allowing for the selection of the optimal device and implantation strategy that could lead to an uneventful Transcatheter Valve implantation [32]. 

**Figure 2:**
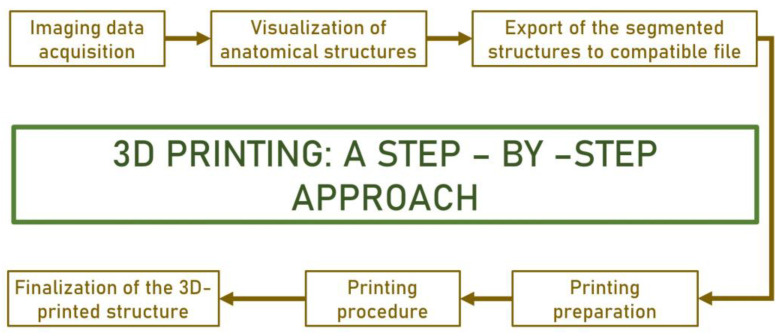
A step-by-step approach for 3D printing.

Additional frequent complications of TAVI are paravalvular leakage (PVL) and prosthesis–patient mismatch [24,33,34,35]. They are associated with worse short-, mid-, and long-term outcomes, thus reducing periprocedural success [24,33,34,35]. For this reason, using the preoperative cardiac CT images, Thorburn et al. created a 3D-printed model with true-size aortic root dimensions, including both the coronary artery ostium location and the left ventricular outflow tract [36], and they implanted a prothesis with a corresponding model and size into the 3D model at an appropriate depth of implantation via fluoroscopy [36]. The evaluation of PVL severity involved utilizing a sealed system with pressurized water infusion lasting for five seconds [36]. The comparison aimed to establish a correlation between the volume measured within the closed-loop model during the pressurized interval and the extent of PVL observed in patients following TAVI, as reported in post-procedural transthoracic echocardiograms [36]. The authors showed a significant association between the degree of PVL reported on postoperative transthoracic echocardiogram and the amount of volume detected in the experimental 3D model, emphasizing the role these 3D models might have in predicting paravalvular leakages [36]. Additionally, Reiff et al. conducted a retrospective analysis of pre-TAVI CT scans for 20 patients with severe, calcific aortic stenosis [37]. Subsequently, 3D aortic root models were printed for each patient using Ninjaflex thermoplastic polyurethane and thermoplastic polyurethane. The models, which were implanted at nominal pressure with same-sized balloon and expandable frames, underwent ex vivo scanning and analysis with Mimics software [37]. Comparison with post-TAVI echocardiograms was performed to assess correlations between identified and predicted PVL locations [37]. Among patients exhibiting echocardiographic paravalvular leak, analyses of ex vivo transcatheter aortic valve replacement 3D models accurately located the paravalvular leak site in eight out of ten cases. Conversely, in patients without paravalvular leak diagnosed with echocardiography, ex vivo TAVI 3D model analyses correctly anticipated the absence of PVL in 90% [37]. Quian et al. created patient-specific aortic root phantoms using the tissue, thereby mimicking the 3D printing technique according to preprocedural computed tomography findings [38]. They developed a novel index, the annular bulge index, for evaluation of post-TAVR annular strain unevenness in the phantoms. This indicator outperformed the other known factors associated with paravalvular leakage and achieved a high level of accuracy in predicting post-procedural paravalvular leakage in terms of its occurrence, severity, and location [38] (Figure 2).

3D printing seems to have several applications in the management of post-TAVI paravalvular leakage. Recently, Aigner and colleagues utilized 3D printing technologies to evaluate flow patterns and hemodynamics of PVLs following TAVI by exploiting 4D flow MRI and standard ultrasound [39]. A physiologically accurate, MR-compatible model of the left ventricle, aortic root, and ascending aorta was crafted through a combination of 3D-printed components and diverse soft silicone materials [39]. Utilizing an Abbott Portico™ valve, the model underwent continuous and retrograde flows, varying transvalvular pressures, and physiological pulsatile hemodynamics for comprehensive time-resolved magnetic resonance measurements, which were subsequently compared to color Doppler ultrasound measurements within an identical experimental setup [39]. Continuous antegrade flow measurements from MRI closely concurred with flowmeter measurements, displaying a maximum error of merely 7%, while in the retrograde configuration, magnetic resonance visualization detected paravalvular leaks with a potential flow overestimation of up to 33% [39]. In the retrograde system, paravalvular leak visualization was feasible from CMR findings, but the flow was overestimated by up to 33% [39]. Additionally, Mao et al. described a case of a 66-year-old male patient having undergone TAVI two years ago who developed severe paravalvular regurgitation (three bundles, volume 11.0 mL). Due to the prohibitive surgical risk, the patient underwent transcatheter closure of the paravalvular leakage using preprocedural guidance with 3D printing and intraprocedural guidance with angiography [40]. Postoperative echocardiography showed that the paravalvular leak was significantly reduced, which confirmed the findings from 3D modeling [40].

Bicuspid aortic valve (BAV) is a common congenital cardiac anomaly associated with elevated risks of aortic complications and valve dysfunction [41]. The elliptical shape of the bicuspid annulus historically excluded BAV patients from TAVI clinical trials, and early TAVI attempts in BAVs reported complications like paravalvular leakage (PVL) [42]. Recent advancements, particularly with the Edwards SAPIEN 3 Ultra valve, show promising results in addressing BAV challenges [43]. Computational modeling, including fluid–solid interaction (FSI), aids in simulating TAVI deployment and assessing PVL, contributing to improved device design and patient outcomes [43]. Complex modeling approaches, such as those by Lavon et al., simulate TAVI in calcified BAVs, evaluating PVL, and stent expansion. These advancements suggest a potential increase in TAVI’s applicability for stenotic BAVs, thus emphasizing ongoing research in this evolving field [44].

Coronary obstruction comprises rare but serious complications following aortic valve replacement. Despite the development of state-of-the-art prostheses, coronary obstruction rates remain high. Heitkemper et al. and Young et al. demonstrated the preoperative reconstruction of the aortic root using 3D printing to analyze calcification distribution, thus aiming to prevent coronary obstruction [45,46]. In high-risk patients, 3D-printed aortic root models allow for in vitro simulation of coronary obstruction incidents. Various balloon sizes are employed to expand the aortic root to comprehensively assess interactions among leaflets, sinuses, coronary openings, and sinus capacity post-balloon dilation, thereby mitigating the risk of coronary obstruction. Additionally, a representative stented valve in the 3D-printed model facilitates observation of the relationship between the stent and coronary openings following device release [10,47]. Russo et al. performed 3D printing in 13 patients with severe AoS with high-risk features for coronary obstruction planned for TAVI. This proof-of-concept study successfully demonstrated that 3D-printed cardiac models may be utilized as a simulation platform in high-risk TAVI cases to assess for preventing coronary obstruction [48].

Furthermore, it is important to predict left ventricle remodeling after TAVI [49,50]. An advanced soft robotic model designed to replicate the patient-individualized hemodynamics associated with aortic valve stenosis and the ensuing ventricular remodeling has recently been introduced [51]. This model integrates 3D-printed replicas of cardiac structures with customized soft robotic sleeves individualized to each particular patient’s hemodynamic characteristics. Two special sleeves were used, an aortic sleeve and a left ventricular sleeve, which mimics the loss of ventricular compliance and diastolic dysfunction. Validated through echocardiographic and catheterization techniques, this model offers superior controllability compared to rigid systems, ensuring a physiological representation of cardiac function. In addition, this model was tested in different backgrounds, presenting consistent results in the assessment of the hemodynamic efficacy of TAVI [52].

In a 2019 Chinese study, researchers explored the postoperative and postmortem applications of patient-specific 3D valve modeling in TAVI complications [53]. Four patients, who experienced post-procedural fatal complications due to device failure despite normal preprocedural CT angiography (CTA), were selected. Using the CTA data, 3D models were created with a 2 mm shell around the blood volume and a 1 mm valve with calcifications. Printed with flexible material mimicking aortic vessel properties, non-valved stent frames were deployed into the patient-specific 3D prints. The outcomes—coronary ostium obstruction or aortic annulus rupture—matched the events seen during TAVI in each patient. The study suggested that preoperative, patient-specific 3D models could aid in predicting adverse outcomes, serving as a valuable supplement to diagnostic imaging for informed treatment planning and care [53].

### 2.2. Mitral Valve

It is estimated that about 2 million individuals in the United States suffer from MV regurgitation, making it the most prevalent valvular disease in the US and the second one globally [14,15,16]. Its prevalence increases with age, accounting for more than 10% in patients >75 years. Its life-long complications affect their quality of life and are responsible for high mortality rates [14,15,16]. For many decades, surgical treatment, either repair or replacement, was the only solution for MV management. Nowadays, transcatheter interventions provide additional therapeutic options for high-risk patients for surgical intervention.

Due to the complexity of transcatheter interventions for mitral valve repair, numerous studies have attempted 3D printing application in mitral devices [54]. After the first successful attempts in 3D printing in healthy and diseased mitral valves, Vukicevic and colleagues prepared a 3D-printed model for the mitral valve device appropriate for in vitro simulation and preprocedural planning for severe mitral regurgitation transcatheter management using the Mitra—Clip Device [55,56,57]. Recently, a novel, 3D-printed simulator, focusing on reproducing the anatomy and plasticity of all areas of the left heart associated with the mitral valve apparatus, was created. The investigators aimed to create a realistic model, where different periprocedural aspects, such as vascular access, transseptal puncture, catheter movements, and leaflet grasping, will be feasible [58].

Additionally, Daemen et al. prospectively modelled the mitral valves of ten patients with various pathologies who had an indication for surgical treatment [59]. In six of them, a 3D-printed, plastic, rigid MV was created for procedural planning, while in the rest of them, they constructed a silicone-cast replica for procedural simulation [59]. The construction time for the rigid plastic valve was about 3–4 h and had a cost between EUR 15 to EUR 40, while silicone models required 2–3 days and their production was more expensive, as it required about EUR 300 [59]. This study showed that 3D printing could be feasible for surgery planning, shortly available and inexpensive [59].

Another option for percutaneous management of mitral valve regurgitation is transcatheter mitral valve replacement (TMVR). In TMVR, the understanding of the “neo” LVOT (left ventricular outflow tract) is crucial for achieving optimal results [60,61]. Nowadays, 3D printing contributes to better comprehension of patient-specific anatomy. A tangible 3D print functions as a communication tool and provides a visual assessment of the intended landing zone for the device within the patient’s mitral plane. Additionally, it provides a visual representation of the predicted size of the “neo” LVOT. Following the acquisition of pre- and post-TMVR procedural CT scans, the concept of the neo LVOT progressed from the physical 3D print to a virtual representation, allowing for valve implantation and simulation using the transcatheter devices of interest [55,62].

After mitral valve implantation, paravalvular leaks are common and associated with adverse events; thus, transcatheter closure using specially developed devices has gained significant ground. Nevertheless, the results of the interventional approach are not always optimal [63]. Due to this problem, Jedrzejek et al. prepared 3D-printed models based on images acquired from TEE and used them for preprocedural planning of transcatheter paravalvular leak management. It was reported that this method could become a helpful tool for such procedures [64]. Further reports have validated 3D printing for the preprocedural planning of transcatheter management of paravalvular leaks in MV [65,66].

Personalized approaches to mitral valve disease and surgical approaches have not been the only approaches supported by 3D printing; several attempts have been made to construct patient-specific devices. Due to the significant variability in the size and morphology of the mitral annulus, one specific ring size and shape is not always suitable for all patients with severe MR. Thus, Pitsis and his colleagues constructed a personalized ring in the operating room with a Dacron sheet and titanium ligating clips to properly fit each individual’s mitral annulus shape and size [67]. In this randomized control trial, fifty-eight patients were treated with the personalized ring, and sixty-nine patients received a commercial Carpentier-Edwards Physio II ring [67]. The results were promising for the novel device; the mitral valve area was greater in the experimental group (3.78 ± 0.22 versus 3.13 ± 0.21, *p* < 0.05) and changed in the course of the cardiac cycle [67]. However, during the eight-year follow-up, no significant difference was observed between the two groups regarding mortality, post-procedural MR severity, or clinical status [67]. Acknowledging the importance of a patient-specific approach in mitral valve regurgitation, Frishman et al. presented a novel device (DynaRing). It is a selectively compliant annuloplasty ring constructed using elastomer segments of various thicknesses, a nitinol core, and a cross diameter filament. Preliminary results have shown DynaRing’s capability to adapt to both anterior–posterior and inter-commissural diameters; thus, it could fit in a wide range of different mitral valves [68]. However, larger studies for further validation are required.

Although mitral regurgitation is the primary MV disease in developed countries, there are still patients suffering from severe calcified rheumatic native mitral stenosis, where 3D printing has also been explored. Zhai et al. described a patient with severe MV stenosis where 3D printing was used to monitor anatomical structures and simulate the intervention. Interestingly, 3D printing changed the physicians’ therapeutic plan as, because of it, interventional and not surgical treatment was preferred; they performed a transcatheter intervention and successfully implanted a balloon-expandable valve in the MV position [69].

### 2.3. Tricuspid Valve

Tricuspid regurgitation (TR) ranks second among valvular pathologies in frequency [70]. In the US, an estimated 1.6 million individuals have TR, with 250,000 new diagnoses annually, yet fewer than 8000 surgeries are performed. Despite its economic burden, with higher hospitalization rates and healthcare-associated expenditure, TR interventions have remained low historically, although recent studies indicate an increasing number of TR surgeries [71]. The 2021 ESC/EACTS guidelines for valvular diseases recommend surgery and medical therapy for TR management, acknowledging the valve’s historical neglect [72]. Surgical treatment, however, carries high periprocedural and in-hospital fatality rates (8.2% to 27.6%) attributed to patients being referred at advanced disease stages [73]. Despite guideline suggestions, surgery remains underused, emphasizing the need for increased awareness and intervention for this prevalent valvular pathology [71]. Against this background, different approaches and devices have been utilized. Transcatheter edge-to-edge repair, transcatheter tricuspid valve replacement, annuloplasty, and heterotopic caval valve implantation have been performed by interventional cardiologists [74]. Although edge-to-edge repair seems to be a step in front of the other methods, its results remain suboptimal.

Amerini et al. were the first to try a personalized prosthesis for the tricuspid valve in porcine models [75]. In this preparatory study, a tridimensional reconstruction of the right-sided cardiac cavities of a 65 kg female pig was obtained in silico. A solid alumide (a material used in 3D printing consisting of nylon filled with aluminum dust) mold was created through rapid prototyping to replicate the inner volume of the right atrium, excluding the auricle. A compressible nitinol stent, designed based on the alumide model, provided stability to biological valve prostheses by transmitting force from the annulus to the atrial wall and the adjacent venae cavae. Several prototypes were developed iteratively, with the initial prototype (P1) named the “bicaval hollow body”. P1 included both venae cavae, coated with a thin silicon layer for sealing. A custom-tailored valve, crafted from porcine pericardium on a nitinol stent, was added. External valve size was 30 mm. P1, and the valve-carrying component could be crimped to a diameter of 8 mm. After primary testing, the second prototype (P2) was created by eliminating the inferior caval component, resulting in a “monocaval hollow body”. P1 and P2 components were connected before implantation, streamlining the procedure to a single-step insertion. The insertion procedure evolved through iterative improvements. Initially, the right atriotomy was performed, and the superior vena cava was snared for total cardiopulmonary bypass. Later cases used fluoroscopy guidance. In the last P1 case, the atrium was opened after insertion, and the valve-carrying component was connected under direct vision. For effective anticoagulation, heparin was partially antagonized after decannulation. The chest was closed, and animals were under observation after cardiopulmonary bypass for approximately two hours. P2, already equipped with its valve-carrying component, was advanced towards the annulus without opening the atrium in subsequent procedures. After the procedure, ten out of twelve animals successfully recovered from cardiopulmonary bypass after device implantation. Successful cases showed stable hemodynamics, no central venous pressure changes, and preserved cardiac rhythm. Echocardiography confirmed proper device positioning and dynamic prosthetic valve function, and postoperative CT scans revealed satisfactory stent fitting without structural deformation. Postmortem examinations revealed no significant lesions or obstruction to the right ventricular outflow tract. This innovative device offers a new therapeutic approach for tricuspid regurgitation because the stent structure lines the right atrium and venae cavae, guaranteeing widespread force distribution from the prosthetic valve. This approach is crucial for patients with advanced tricuspid regurgitation; the variable dimensions of the right atrium make standardized sizing unfeasible. Harb and et al. have utilized 3D printing for optimizing surgical management of primary, severe TR. They presented four cases where 3D printing was fundamental to procedural planning as well as patients’ better education [76].

3D printing also has applications in the transcatheter management of TR [77,78,79]. Mao et al. used 3D printing for the preprocedural evaluation of transcatheter treatment of severe lead-induced TR [80]. Prior to transcatheter tricuspid valve replacement (TTVR), a bench test involved assessing the 3D-printed TV model. LuX-Valve implantation was guided by TEE and X-ray fluoroscopy, with transatrial access employed for all patients [81]. The implantation was successful in all six patients, with two-year echocardiographic follow-up revealing no or trace in five patients and mild in one; thus, 3D printing might play a crucial role in the personalization of transcatheter TR management in the near future [80]. Similar efforts using 3D printing for preprocedural guidance have been performed using the transjugular tricuspid valve-in-ring replacement and the LuX-Valve Plus system for failed annuloplasty ring cases [77,82].

## 3. Left Atrial Appendage Occlusion (LAAO)

Left atrial appendage occlusion has gained ground in the management of high-bleeding-risk patients with atrial fibrillation suffering from recurrent strokes. Such patients have multiple comorbidities, such as heart failure or kidney disease, and their management is challenging [83,84,85]. Its safety and efficacy have been validated in numerous randomized clinical trials, and its utilization in clinical practice is increasing steadily [86,87,88]. Careful preprocedural planning and optimal multimodality imaging are crucial; thus, both transesophageal echocardiography and multidetector computed tomography, which acquires a 3D dataset, an improved spatial resolution, and unlimited reconstruction, are performed. Despite the significant progress, the complex morphology and the special anatomy of each individual may drive device failure and suboptimal results [89]. Current data have shown that moderate or severe peri-device leaks exist in 10–27.5% of patients following the procedure. These reports not only indicate the possible adverse events that could potentially arise from PVL, but also report the emerging issue/problem of suboptimal sizing [87,90].

Against this background, Otton and colleagues were the first to utilize 3D printing in LAA closure for choosing the optimal device size, thereby assessing the impact of the anatomic deformation of each device. Surprisingly, the sizing made by 2D TEE was not in accordance with 3D printing sizing [91].

Conti et al. evaluated six patients with diagnosed LAA leak and fourteen patients without leak after transcatheter occlusion, as it has been estimated with TEE. According to CT preoperative images, a patient-specific 3D model was produced, and a size suggested by the 3D model was compared with the size of the implanted device. They showed that about half of the devices’ sizes were underestimated, and only in 35% were the two sizing methods in accordance. The incidence of LAA leak was significantly higher in the patients with an underestimated device size compared to the rest of the patients [92]. DeCampos and colleagues have performed a meta-analysis, including eight studies and 283 patients, comparing an imaging-only with a 3D printing approach [93]. Despite the small sample and the lack of randomized data, the 3D printing approach significantly reduced peri-device leak, showing the road to further randomized trials.

## 4. 3D Printing Cost Analysis and Effectiveness

The implementation of 3D printing strategies in healthcare may face challenges due to high start-up costs, including the need for equipped space, advanced devices, and specialized personnel, consisting of a minimum of three specialists (Attending Physician, Fellow Cardiologist, and Engineer). Printing costs can be influenced by various factors, such as object weight, printing time, electricity tariff, and filament cost. For instance, the total process time from order initiation to print completion of 3D models in cardiology ranges from 7.6 h for low-resource models to 19.3 h for high-resource ones, with costs varying from USD 717.19 to USD 1157.85, respectively. However, despite these initial hurdles, the benefits of 3D printing technology in healthcare are significant. Personalized medical devices and equipment produced by 3D printers can lead to notable cost savings in overall medical expenses. Custom-made devices and low-cost surgical tools can improve surgical outcomes, reduce hospital stays, and minimize treatment failure costs. Moreover, by streamlining manufacturing processes and reducing unnecessary material usage, 3D printing offers higher speed and rapid production of medical products, contributing to cost efficiency in healthcare institutions [94,95].

## 5. Ethical Considerations and Legal Issues for 3D Printing

The proliferation of 3D printing technology has revolutionized medical treatments and opened new frontiers in numerous specialties. However, alongside its remarkable potential, 3D bioprinting presents several ethical considerations that must be addressed as these technologies advance and legal frameworks must soon be established.

First, the widespread adoption of 3D printing has the potential to exacerbate existing disparities in healthcare accessibility. The costs associated with this technology and its related services may be prohibitive for certain communities, thereby widening the gap in access to advanced medical treatments. Secondly, ethical dilemmas arise concerning the utilization and safeguarding of sensitive patient data. Concerns revolve around the security and privacy of these data, as any breaches or misuse could compromise patient confidentiality and diminish trust in healthcare systems [96].

Moreover, obtaining informed consent from patients becomes increasingly intricate. Patients must fully understand the risks, benefits, and uncertainties associated with these personalized treatments before they make medical decisions. Given the complexity of such technologies, healthcare providers must devise comprehensive strategies to ensure sufficient patient education and empowerment during the informed consent process. Furthermore, the potential for errors during the manufacturing process poses a significant concern. Ensuring the safety and reliability of 3D-printed medical products becomes paramount, as any defects or inaccuracies could have severe consequences for patient health and well-being [97].

Lastly, issues concerning intellectual property rights, commercialization of bioprinted parts, and government intervention in research and technology development are some of the most important legal considerations surrounding 3D printing. While bioprinted parts may be treated as objects of civil rights before transplantation, legal recognition of ownership termination upon implantation is necessary. Existing laws may not adequately address the unique nature of bioprinted organs, necessitating the development of a comprehensive legal framework. Government intervention is crucial in determining the future trajectory of 3D bioprinting technology to ensure ethical and responsible use [98].

Overall, these considerations represent only an initial estimation of the burgeoning ethical, legal, and social problems expected to arise with the progression and widespread use of 3D bioprinting technology [99]. Therefore, we could conclude that the evolving landscape of 3D bioprinting applications holds the potential not only to offer improved treatment options but also to confront an expanding array of ethical dilemmas, which require the advent of international regulatory directives.

## 6. Limitations

Our review has several limitations that should be addressed. First of all, it is not a systematic review but rather a narrative review of the literature. Secondly, there is a lack of empirical data from randomized clinical trials or similarly well-designed comparative studies and comprehensive data analyses to provide evidence for the superiority of the new approach; thus, the ability to assess the direct impact of the technology on treatment outcomes is limited. Moreover, we did not discuss the long-term effectiveness and safety of using 3D models in cardiovascular medicine, as no relevant studies exist due to the recent introduction of 3D modeling in clinical practice.

## 7. Conclusions

In conclusion, personalized applications of 3D printing in interventional cardiology and cardiac surgery represent a transformative paradigm in the management of structural heart diseases. This review underscores the pivotal role of 3D printing in enhancing procedural precision, from preoperative planning to procedural simulation, particularly in valvular heart diseases, such as aortic stenosis and mitral regurgitation. The ability to create patient-specific models contributes significantly to predicting and preventing complications like paravalvular leakage, ensuring optimal device selection, and improving outcomes. Additionally, 3D printing extends its impact beyond valvular diseases to tricuspid regurgitation and non-valvular structural heart conditions. The comprehensive synthesis of the existing literature presented here emphasizes the promising trajectory of individualized approaches facilitated by 3D printing, promising a future where tailored interventions based on precise anatomical considerations become standard practice in cardiovascular care.

## Data Availability

Not applicable.
